# Some Comparatively New Facts about an Old Remedy—The Muriated Tincture of Iron

**Published:** 1893-03

**Authors:** E. F. Starr

**Affiliations:** Nacoochee, Ga.


### SOME COMPARATIVELY NEW FACTS ABOUT AN OLD REMEDY—THE MURIATED TINCTURE OF IRON.

By E. F. STARR, M. D.,
NACOOCHEE, GA.

This medicine has grown somewhat old in the hands of the profession and it may not be the only remedial agent which has done so, without having been utilized to the full extent of its capabilities. Indeed, there are few of the old established remedies that might not be made to develop something new, by close study and careful manipulation, and it is well known that the value of a good medicine depends very much upon the manner in which it is handled. I am for improvements and discoveries when truthfully so denominated, but in this day of vastly accumulated and still accumulating new remedies, to an extent beyond the power of memory to retain, it may be restful at times to have the attention diverted in another direction. The tincture of iron has some capabilities which have not been universally recognized. Its astringent properties are more familiar now than formerly. The late Dr. W. F. Westmoreland had great confidence in it in surgical practice as an astringent and hemostatic. It is certainly a powerful astringent and is thoroughly antiseptic. Clots of blood that are formed by coming in contact with it, or any that are imbued with it, are firmly set against septicism and resist putrefaction for a long time. But this fluid is not only a local and traumatic hemostatic, for when administered internally it sometimes produces hemostatic effects. For instance, a woman of fifty years of age was bleeding at the lungs and sent to me for advice. I prescribed forty drops of tincture of iron to be taken in water and repeated as might be necessary. She was relieved and is living now, years after.

A young physician, probably twenty-five years of age, had nephritic hemorrhage. His urine was considerably colored with and sometimes consisted in a large measure of fluid blood. He tried various astringents, such as tannic acid, gallic acid, lead and zinc; cholagogue cathartics were also used, but the waste continued. He then took forty drop doses of tincture of iron, which promptly arrested the flow without producing disagreeable effects, and since that, having had one or more visitations, he has been promptly relieved by the same medicine.

I have used from a fourth to an eighth solution of this medicine with water with happy effect, for nasal catarrh and for abraded surfaces generally. I have found few things so remedial and healing in these conditions as this preparation. In nasal catarrh from a few drops to a teaspoonful may be poured into the nostrils with the head turned back so as to allow the solution to descend through the nasal canal and thus come in contact with the surfaces and find an egress at the pharynx.

But of more importance is the tincture of iron as a remedy for scalds and burns. We all know something of the intensity of the pain and suffering produced by these accidents, and the agonies and troublesome character of the sores they produce make strong demand for prompt relief and a reliable treatment. Happily now, we have the means for such relief in the muriated tincture of iron. Of course there may be burns so deep and severe that *prompt* relief cannot be expected, but for all ordinary occasions and even for the worst it may be resorted to with confidence in its beneficial effects. Much depends upon the promptness of its use, but when instantaneous promptness is not practicable let it be resorted to nevertheless.

Immediately, or at the earliest possibility after the occurrence, apply the tincture of iron over the surface of the burn with a feather or soft brush, so as to moisten it everywhere. Where the cuticle is not destroyed and removed it should be used full strong; if the cuticle is gone and the surface raw, dilute with water one-half or two-thirds. How prompt the relief from pain is after the application no one can tell save one who has tried it. In scalds and superficial burns the immediate application will not only allay the pain but prevent blistering. There is a characteristic feature connected with burns that deserves consideration, and that is the tendency to too much and too long continued suppuration—to become chronic and to continue indefinitely. Old sores from burns have usually been hard to cure up. For the relief of these kind of traumatisms the proper course to pursue is to prepare and use a salve in the following way: Take of vaseline or lard one ounce, of tincture of iron one drachm, more or less, combine the two by rubbing together either in a mortar or in a plate or saucer with a knife or spatula. Spread this salve thinly upon a soft cloth and apply to the entire raw surface; this will soon diminish the flow from the raw surface and cause it to heal rapidly.

There is another class of diseases, very important, pertaining to the realm of gynecology in which this medicine may be used with great benefit. The credit for its introduction in this line of practice, so far as I know, is due to the late John M. Johnson, of Atlanta, to whom a debt of gratitude should be considered due, both from the profession and from suffering females. From the profession because it is supplied with an efficient method of treating a troublesome and sometimes perplexing condition; and from females because they may be the beneficiaries of an efficient treatment, and also because they may manipulate most of the treatment for themselves, and thus be saved the trouble, annoyance and trial of delicacy incident to frequent specular examinations by the physicians. I allude to vaginal and uterine leucorrhœa, and especially the latter, which is a symptom of a condition, and that condition is chronic or superacute inflammation of the mouth and inner surface of the neck, and if long continued and aggravated, extends to the substance of the wall itself, and involving the condition of hyperplasia, as designated by some. The vaginal and uterine leucorrhœa may prevail at the same time, or each one may exist without the other. My favorite remedy is the muriated tincture of iron. Among nice people uterine leucorrhœa seldom occurs, except as a result of the condition of subinvolution, and with a little experience this condition is easily detected by the rational symptoms. First, there is the old story, "I have not been well since my last child was born," and then there is leucorrhœa all the time, and increasing rather than getting better; there is constant, or almost constant, backache, there are hot flashes and flushed cheeks, alternate cold and hot feet and hands, there is depression of spirits, loss of appetite, debility and nervous troubles without number and without end, and add to these general malaise, vain imaginings, and now and then hysterical exacerbations. For treating a case of uterine leucorrhœa and subinvolution, one part of tincture of iron should be diluted with two parts of water and used as an injection or vaginal wash with a glass female syringe twice a day, or night and morning. The instrument need not be entirely filled, half full, or the amount of a tablespoonful, is enough to use at a time. Its use should be continued daily until there is a feeling of soreness or tenderness produced in the parts and the beginning of an exfoliation from the inner surface of a darkened or black color and representing the cast off surface to which the medicine has been applied. When this begins to take place the use of the medicated injection should be discontinued until the exfoliation is completed, which may be assisted by the use of warm water with the common syringe, or the warm water douche. After the exfoliation is completed, or after a rest of three or four days from the iron injections, a similar course should be commenced again and continued until a similar effect is produced. Usually time may be found for two courses of this treatment between menstrual periods, or during each month; of course it must not be continued nor commenced during a menstrual period, as this function when being normally performed should be scrupulously respected. When the method above described of utilizing this medicine has been pursued a sufficient time, with other adjuvants to suit the general symptoms, the patient will be found improving in general health, with a subsidence of the local symptoms. This is not all that might be said, but with study and trial the muriatic tincture of iron is capable of extensive adaptability.

